# Supplementation with Beef Extract Improves Exercise Performance and Reduces Post-Exercise Fatigue Independent of Gut Microbiota

**DOI:** 10.3390/nu10111740

**Published:** 2018-11-12

**Authors:** Tsung-Hsien Hsu, Chien-Chao Chiu, Yu-Chih Wang, Ter-Hsin Chen, Yi-Hsun Chen, Yen-Peng Lee, Shao-Wen Hung, Chean-Ping Wu, Hsiao-Li Chuang

**Affiliations:** 1 Division of Animal Industry, Animal Technology Laboratories, Agricultural Technology Research Institute, Miaoli 350, Taiwan; tzong@mail.atri.org.tw (T.-H.H.); chiu2295@gmail.com (C.-C.C.); 1032169@mail.atri.org.tw (S.-W.H.); 2 Department of Animal Science, National Chiayi University, Chiayi 600, Taiwan; wcp@mail.ncyu.edu.tw; 3 Graduate Institute of Veterinary Pathobiology, National Chung Hsin University, Taichung 402, Taiwan; ycw2017@email.nchu.edu.tw (Y.-C.W.); thc@dragon.nchu.edu.tw (T.-H.C.); u9423201@gmail.com (Y.-H.C.); zoosupreme@gmail.com (Y.-P.L.); 4 National Laboratory Animal Center, National Applied Research Laboratories Research Institute, P.O. Box 86 Academia Sinica, Taipei 115, Taiwan

**Keywords:** beef extract, exercise performance, antifatigue, swimming, glycogen

## Abstract

Beef extract (BE) is a nutritional supplement obtained by cooking beef meat. Compared with traditional chicken essence or clam extract, BE is cheaper to produce and may be used for wound healing, as a chemotherapy supplement, or to prevent fatigue. In this study, we evaluated the potential beneficial effects of BE on exercise performance and the related role of the gut microbiota. Pathogen-free male BALB/c mice were divided into three groups to receive vehicle or BE (0, 12.3, or 24.6 mL/kg) by oral gavage for 28 days. Exercise performance was evaluated using forelimb grip strength, swimming time to exhaustion, and physiological levels of fatigue-related biomarkers (serum lactate, blood urea nitrogen, and glucose levels) after physical challenges. BE supplementation elevated endurance and grip strength in a dose-dependent manner; significantly decreased lactate and blood urea nitrogen levels after physical challenge; and significantly increased muscle glycogen content. The germ-free mice supplemented with BE or an equal-calorie portion of albumin did not show significant differences from the other groups in exercise performance and levels of related biomarkers. Therefore, BE supplementation improved endurance and reduced fatigue, which might be related to BE composition, but had no correlation with the gut microbiota.

## 1. Introduction

Beef extract (BE) is a liquid nutritional supplement obtained by cooking beef meat. This supplement has been used for strengthening the bones and muscles, and for increasing immunity [1,2,3]. Compared with other kinds of meat, beef contains higher amounts of amino acids and trace elements, including vitamin B6, vitamin B12, iron, and zinc, which can be extracted into a broth or soup, thereby improving the absorbability of these nutrients by the body [4].

Previous reports showed that supplementation with beef-related foods such as powder-hydrolyzed beef protein, whey protein, and micellar casein enhanced exercise performance [5,6]. Moreover, dose-dependent myofibrillar protein synthesis resulting from beef ingestion was enhanced with resistance exercise in middle-aged men [7], and BE supplementation increased the relative weights of both the soleus and extensor digitorum longus muscles in rats [1]. BE contains large amounts of physiologically active substances such as l-carnitine. l-carnitine plays an important role in fat metabolism in that it promotes the mitochondrial uptake of long-chain fatty acids for β-oxidation coupled with adenosine triphosphate (ATP) production [8]. This finding suggests that the soleus muscle could effectively utilize the ATP energy produced by β-oxidation of fatty acids, which results in reduced consumption of stored glycogen [9].

Exercise-induced fatigue can have deleterious effects on physical activities, quality of life, and social relationships. Many studies have demonstrated that muscle damage and delayed-onset muscle soreness can result in loss of muscle force and significant fatigue [10,11,12]. Muscle soreness and damage represent considerable obstacles to exercise performance. Currently, the role of inflammation-related metabolites such as creatine kinase, lactate, and ammonia in muscle damage after heavy exertion is well documented [13,14]. After exercise, skeletal muscle fatigue was evaluated using biochemical indicators, including lactate, and blood urea nitrogen (BUN) level increased and glucose (GLU) level decreased in serum [5,15]. Furthermore, glycogen is the predominant source of glycolysis for ATP production. Therefore, the amount of stored glycogen in the muscle directly affects exercise ability [15]. Long-term endurance exercise may induce muscular injury [16,17], and muscle damage combined with the production of inflammatory mediators from exhaustive exercise may lead to increased pain and performance deficits in muscle function [18]. The beneficial effect of chicken essence or branched chain amino acid (BCAA) supplementation on physical fatigue has been reported. Therefore, we determined whether BE supplementation could help prevent fatigue and improve exercise performance.

The human and animal intestinal tract contains approximately 100 trillion microbes, most of which reside in the colon [19]. Recent reports indicate a close correlation between the efficacy of nutritional supplements and the host gut microbiota [20,21,22]. A particularly compelling example of the importance of the gut microbiota in host metabolism is provided by a comparison of the nutritional statuses of germ-free (GF) and conventionally raised laboratory animals [23]. Numerous investigators have demonstrated that conventionally raised animals require up to 30% less caloric intake to maintain their body weight. In addition, our previous study also demonstrated that the gut microbiota can affect endurance in a GF mouse model [24]. This was proposed to occur through the action of the gut microbiota on host antioxidant enzyme systems [24].

Our study elucidated the beneficial effects of 28-day BE supplementation on exercise performance and fatigue and showed that its sub-acute effects raised no health concerns requiring attention, and that gut microbiota might not play a pivotal role in the metabolism of BE supplements.

## 2. Materials and Methods

### 2.1. Preparation of BE

Beef meat to be used for supplementation was purchased from the local market. The meat from Taiwan yellow cattle was cut into 5-cm^3^ pieces and cooked at 100 °C for 10 h. The crude BE was stored at −80 °C until use. The nutritional facts and total branched-chain amino acids of the BE were analyzed by SGS Taiwan Ltd. (SGS Taiwan limited, New Taipei, Taiwan) and are shown in Table 1.

### 2.2. Animal Experiment Design

Specific pathogen-free (SPF) male BALB/c mice were purchased from BioLASCO Technology (Taipei, Taiwan), and germ-free (GF) male BALB/c mice were purchased from the National Laboratory Animal Center (Taipei, Taiwan). Mice were used for the experiments at 7 weeks of age. Prior to starting the experiments, the SPF and GF mice were housed for 1 week to adapt to the environment. The GF mice were housed in sterilized isolator caging. All the animals were kept at room temperature (21 °C ± 2 °C), with 55–65% relative humidity and a 12-h light-dark cycle. The mice were fed a standard laboratory rodent diet (5010 LabDiet, Purina Mills, St. Louis, MO, USA) and provided with water ad libitum. All the animal experiments conformed to the guidelines of the Institutional Animal Care and Use Committee (IACUC) of National Chung Hsing University. This study was approved by the IACUC ethics committee under protocol 106-032. For the first set of experiments, the SPF mice were divided into three groups (*n* = 6–8 per group in each test) as follows: (1) vehicle control, (2) 12.3-mL/kg BE (BE-1X), and (3) 24.6-mL/kg BE (BE-2X). The control group received the vehicle and distilled water at the same dosage volume of 24.6 mL/kg for the same period as the test mice. The mice underwent a grip strength test 1 h after the final administration of the BE supplement on day 25. The mice performed an acute exercise challenge on day 26. The mice performed an exhaustive swimming exercise on day 28. For the second set of experiments, the GF mice were divided into three groups (*n* = 5 per group), namely the vehicle control (GF-veh), albumin (GF-Alb; Sigma-Aldrich, St. Louis, MO, USA), and BE-2X groups (24.6 mL/kg; GF-BE). The vehicle, albumin, and BE were administered via oral gavage. The mice performed an exhaustive swimming exercise on day 28.

### 2.3. Forelimb Grip Strength Test

A low-force testing system (Model-RX-5, Aikoh Engineering, Nagoya, Japan) was used in this test. The tensile force in each mouse was measured using a force transducer equipped with a metal bar (2 mm in diameter and 7.5 cm in length). We grasped the mouse at the base of the tail and lowered it vertically toward the bar. Once the two paws (forelimbs) grasped the bar, the mouse was pulled slightly backward by the tail, which triggered a “counterpull”. The grasping force was recorded by a grip strength meter in grams. Forelimb grip strength was tested after four weeks of administration of the indicated BE supplement.

### 2.4. Swimming Exercise Performance Test

The swimming endurance test was conducted after the forearm grip strength test for all the mice. The mice were individually placed in a columnar swimming pool (height, 30 cm and radius, 10 cm) with a 20-cm water depth, maintained at 27 °C ± 1 °C. The swimming endurance time of each mouse was recorded from the beginning of the test until exhaustion, which was determined by observing loss of coordinated movements and failure to return to the surface within 7 s. Swimming endurance time was determined after four weeks of administration of the indicated BE supplement. The swimming exercise performance tests for all the mice were conducted on the same day as the forelimb grip strength measurement test.

### 2.5. Lactate, BUN, and GLU Levels after Acute Exercise Challenge

The effects of BE supplementation on serum lactate, BUN, and GLU levels were evaluated after exercise. Approximately 1 h after the last administration of BE, a 10-min swimming test was performed. Blood samples were collected from the submandibular duct of the pretreated mice immediately before and after the swimming exercise. Serum was extracted by centrifugation at 2600× *g* and 4 °C for 10 min. Serum lactate concentration was measured using a lactate assay kit (Sigma-Aldrich, St. Louis, MO, USA), and serum BUN and GLU concentrations were determined using an auto-analyzer (Hitachi 7080, Hitachi, Tokyo, Japan).

### 2.6. Clinical Biochemical Analysis after Sacrifice of the Animals

To examine the subchronic toxic effects of BE supplementation, serum alanine aminotransferase (ALT), GLU, BUN, triacylglycerol (TG), and total cholesterol (T-CHO) levels were evaluated after the animals were sacrificed. Whole-blood samples were collected through cardiac puncture and centrifuged at 2600× *g* and 4 °C for 10 min. Serum was immediately stored at −80 °C until analysis, and the biomarkers were determined using an auto-analyzer (HITACHI 7080, Hitachi, Tokyo, Japan).

### 2.7. Tissue Glycogen Level Measurement and Visceral Organ Weight

The stored form of GLU is glycogen, which is primarily stored in the liver and muscle tissues. One hour after the final BE supplementation, all the mice were sacrificed, and the liver and muscle tissues were excised and weighed prior to glycogen content analysis. One hundred milligrams of liver and muscle tissues was homogenized in 0.5 mL of cold 10% perchloric acid. After centrifugation at 15,000× *g* at 4 °C for 15 min, the supernatant was carefully removed and kept on ice prior to analysis. Tissue extract (30 μL) was placed in 96-well microplates, and 200 μL of iodine-potassium iodide reagent was added to each well. An amber-brown compound developed immediately after the reaction. After allowing the plate to rest for 10 min, an enzyme-linked immunosorbent assay reader (Thermo Multiskan GO, Thermo Fisher Scientific, Vantaa, Finland) was used to measure the absorbance.

### 2.8. Histopathological Examination

Mouse tissue samples, including those from the liver, kidneys, muscles, and spleen, were fixed in 10% neutral-buffered formalin for 1 day, dehydrated, embedded in paraffin, cut into 4-μm slices, and stained with hematoxylin and eosin (H&E) for histological examination.

### 2.9. Fecal Short-Chain Fatty Acid Analysis by High-Performance Liquid Chromatography

Fecal samples were collected immediately after defecation. To each fecal sample, 1 mL of 70% methanol was added for extraction and mixed well using a glass stick. The mixtures were centrifuged at 12,000× *g* for 10 min, and the supernatant was collected for analysis. High-performance liquid chromatography (HPLC) was performed using an Agilent 1260 series HPLC (Agilent, Santa Clara, CA, USA) and a YMC column (YMC, Kyoto, Japan). The mobile phase was composed of acetonitrile, methanol, and water at a ratio of 30:16:54, and the pH was adjusted to 4.5 with 0.1% trifluoroacetic acid (special grade; Wako Pure Chemical Industries, Osaka, Japan). The column temperature was 25 °C, and the flow rate was 1 mL/min. Measurements were performed at 400 nm.

### 2.10. Statistical Analysis

Data are presented as mean ± SD. Significant differences between each treated group were determined using one-way analysis of variance and post hoc Fisher least significant differences test using the SPSS 18.0 software (IBM Corporation; Armonk, NY, USA). Differences between the groups were considered statistically significant (*) when their *p* values were <0.05.

## 3. Results

### 3.1. Effects of BE Supplmentation on Body Weight, Skeletal Muscle Mass, Liver Weight, and Clinical Biochemistry

No significant changes in body weight, skeletal muscle weight (comprised of the gastrocnemius and soleus muscles), and liver weight were found among the vehicle, BE-1X, and BE-2X groups (Table 2). As shown in Table 3, no significant differences in the serum concentrations of ALT, GLU, BUN, T-CHO, and TG were found among the groups. These results suggest that the BE supplementation was safe for all the test animals.

### 3.2. Effects of BE Supplementation on Forelimb Grip Strength and Endurance

**Figure 1:**
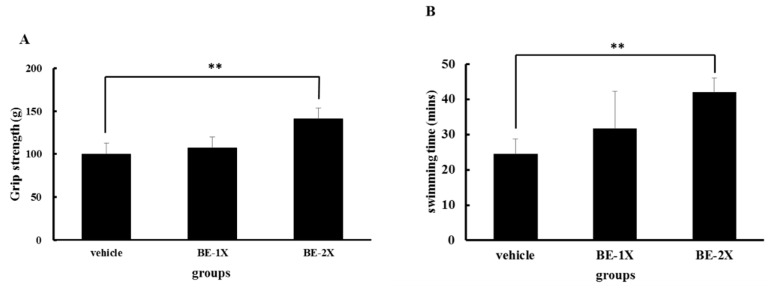
Effect of BE supplementation on forelimb grip strength and swimming exercise performance. (**A**) The mice underwent a grip strength test 1 h after the final administration of BE on day 25. (**B**) The mice performed an exhaustive swimming exercise on day 28. Data are presented as mean ± SD (*n* = 6–8 mice in each group). ** *p* < 0.01, compared with the vehicle control.

The results of the grip strength test were 99.8 ± 11.1, 107.0 ± 13.9, and 141.1 ± 22.8 g for the vehicle, BE-1X, and BE-2X groups, respectively. The grip strength of the BE-2X group was significantly higher than that of the vehicle control (*p* = 0.002). These results showed that BE treatment was beneficial for grip strength (Figure 1A). The exercise endurance of the mice treated with the vehicle, BE-1X, and BE-2X were 24.5 ± 4.2, 31.7 ± 10.6, and 42.0 ± 4.1 min, respectively (Figure 1B). The swimming time was significantly longer with BE-1X and BE-2X supplementation than with vehicle treatment by 1.29- (*p* = 0.097) and 1.71-fold (*p* < 0.001), respectively.

### 3.3. Effects of BE Supplementation on the Serum Levels of Lactate, BUN, and GLU after Acute Exercise Challenge

**Figure 2:**
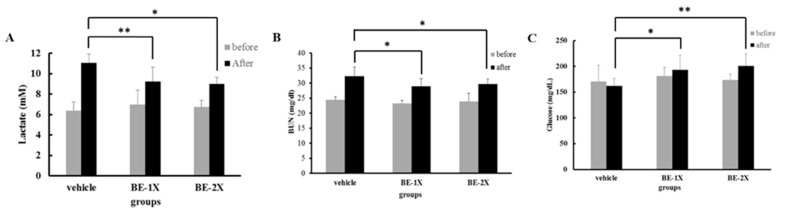
Effect of four-week BE supplementation on serum biomarker concentrations. (**A**) Serum lactate, (**B**) blood urea nitrogen, and (**C**) glucose levels before and after a 10-min swimming exercise challenge. The data are for *n* = 6–8 mice per group and presented as mean ± SD. * *p* < 0.05; ** *p* < 0.01, compared with the vehicle control.

The serum lactate levels in the BE-1X and BE-2X groups were significantly decreased compared with the in the vehicle group (Figure 2A). The serum BUN level in the BE-2X group was significantly decreased as compared with that in the vehicle group (Figure 2B). By contrast, the serum GLU concentration increased with BE-2X supplementation as compared with the vehicle (Figure 2C).

### 3.4. Effects of BE Supplementation on Muscular and Hepatic Glycogen Concentrations

**Figure 3:**
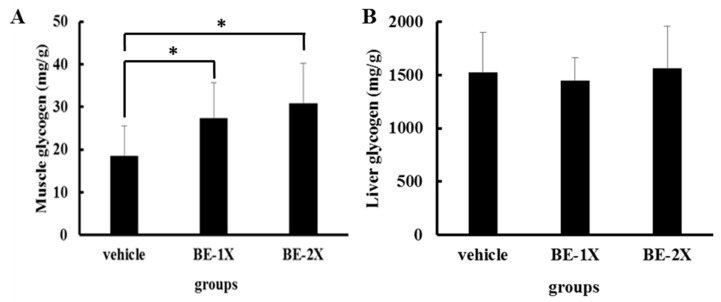
Effects of BE supplementation on (**A**) muscular and (**B**) hepatic glycogen levels. The mice were pretreated with vehicle, 12.3-mL/kg BE (BE-1X), or 24.6-mL/kg BE (BE-2X) for 28 days. Then, the mice were sacrificed, and the glycogen concentrations in muscle and liver tissues 1 h after the final BE treatment were estimated. The data represent the mean ± SD of 6–8 mice in each group. * *p* < 0.05, compared with the vehicle control.

The muscle glycogen levels in the vehicle, BE-1X, and BE-2X groups were 18.06 ± 6.9, 27.4 ± 8.2, and 30.8 ± 9.4 mg/g of muscle, respectively (Figure 3A). A significant increase in muscle glycogen content was observed in the BE-1X (*p* = 0.03) and BE-2X groups (*p* = 0.01) as compared with the controls. Although we did not find any significant differences in hepatic glycogen content among the groups, a slight increase in hepatic glycogen concentration was found in the BE-2X group as compared with the vehicle group (Figure 3B).

### 3.5. Histological Examination after BE Supplementation

**Figure 4:**
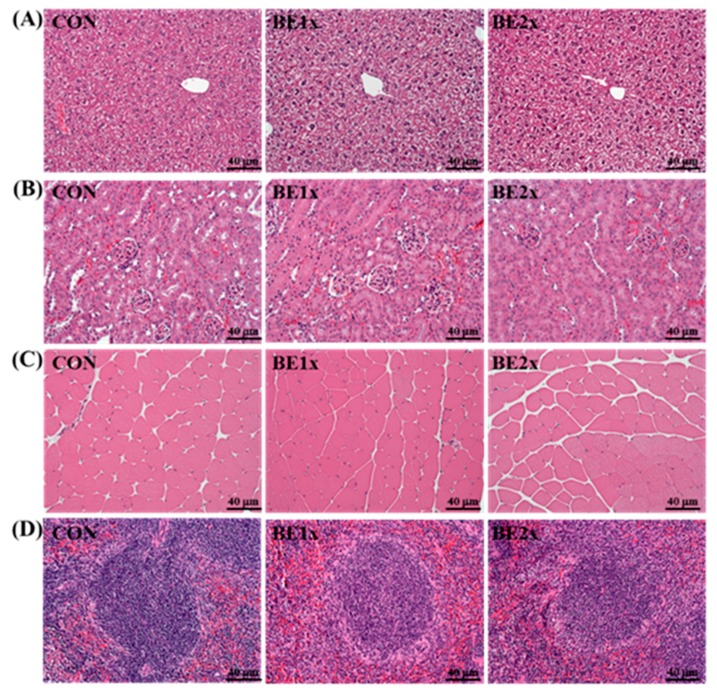
Effects of BE supplementation on the morphology of the (**A**) liver, (**B**) kidney, (**C**) skeletal muscle, and (**D**) spleen. The mice were pretreated with vehicle, 12.3-mL/kg BE (BE-1X), or 24.6-mL/kg BE (BE-2X) for 28 days. Then, the mice were sacrificed, and the liver, kidney, skeletal muscle, and spleen morphologies were examined at the end of the experiment. Hematoxylin and eosin staining: original magnification, ×100 and scale bar, 40 μm.

We considered that BE supplementation for four weeks might have negative or unpredictable effects on healthy mice. Therefore, we evaluated the major organs, including the liver, skeletal muscle, heart, kidneys, and spleen of the mice in each group by histopathological examination. As shown in Figure 4, no adverse effects such as Zenker’s necrosis in muscle or tubular epithelial degeneration in the kidney were observed in any of the groups.

### 3.6. Fecal Short-Chain Fatty Acid Profiles after BE Supplementation

**Figure 5:**
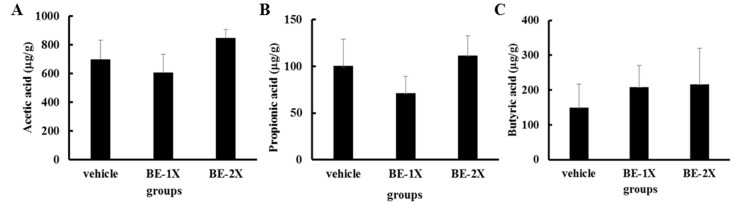
Effect of BE supplementation on fecal short-chain fatty acid content: (**A**) acetic acid, (**B**) propionic acid, and (**C**) butyric acid. Data are expressed as mean ± SD.

We further examined whether BE supplementation at different concentrations might affect the short-chain fatty acid composition of the feces. However, no significant differences in the levels of acetic acid, propionic acid, and butyric acid were observed among the groups (Figure 5).

### 3.7. Improved Exercise Performance after BE Supplementation Is not Associated with the Gut Microbiota

**Figure 6:**
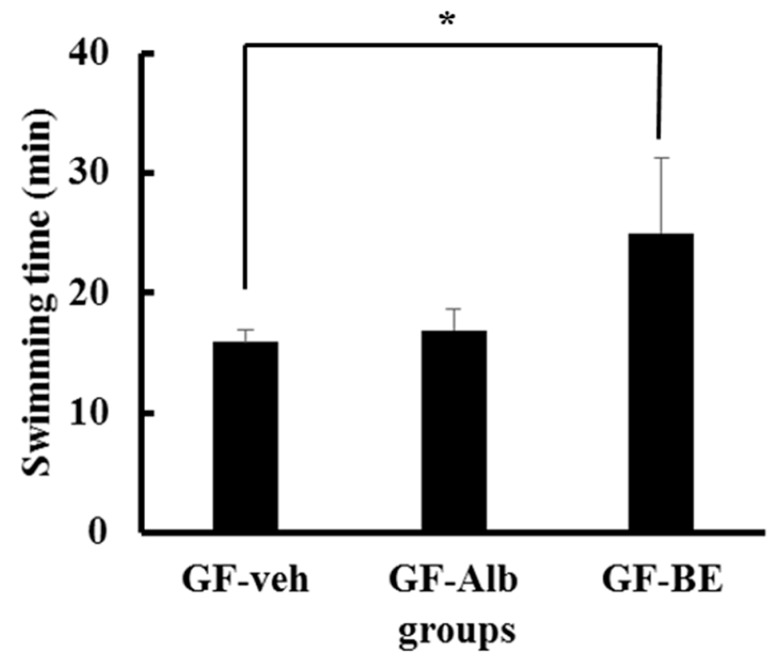
Effect of BE supplementation on the swimming exercise performance of germ-free mice. The mice underwent an exhaustive swimming exercise on day 28 of the study. Data are presented as mean ± SD (*n* = 5 mice in each group). * *p* < 0.05, compared with the vehicle control.

Finally, we clarified whether the gut microbiota plays a role in BE supplementation-enhanced exercise performance. We used the germ-free mouse model to verify that BE supplementation directly affected exercise performance. No significant changes were observed in body weight, skeletal muscle weights (comprised of the gastrocnemius and soleus muscles), and levels of the biochemical analytes (ALT, GLU, BUN, T-CHO, and TG) among the GF-veh, GF-Alb, and GF-BE groups (Table 4). The exercise endurance in the GF-BE group was significantly increased by 1.57-fold as compared with that in the GF-veh group (*p* = 0.028; Figure 6). However, no significant difference in endurance was found between the GF-veh and GF-Alb groups (Figure 6). These results confirmed a previous hypothesis that BE supplementation could directly affect exercise performance.

## 4. Discussion

Recently, protein-rich foods, including chicken essence and clam extract, have been reported to enhance exercise stamina. In the present study, we found that BE supplementation had a similar positive effect on athletic performance by enhancing endurance and reducing muscle fatigue without detrimental effects on the host. Furthermore, in this study, the results, including body weight, skeletal muscle mass, liver weight, clinical biochemistry, and histological examination, after BE supplementation were not significantly different compared with those with the vehicle. The above-mentioned results suggest that BE supplementation is safe for all test animals. Supplementation with BE may improve endurance and performance, even without training, by increasing the potential for athletic exercise. Moreover, we also provided strong evidence that the benefits of BE supplementation were not related to the host microbiota.

Our results showed that supplementation with BE does not affect body growth or enhance skeletal muscle weight. These results differ from those of previous studies that showed that ingestion of beef can increase muscle weight gain in middle-aged men and rats [1,7]. We suspect that the increased muscle volume is dependent on a combination of training such as resistance exercises and beef ingestion. Our results possibly differ from the previous results because only BE supplement was performed in present study. 

Previous studies also suggested that grip strength must be improved through procedural exercise training [25,26]. As shown in Figure 1, grip strength was greater in the BE-2X group than in the control group after 4 weeks of BE supplementation. Thus, in the absence of an additional training intervention, BE supplementation provided only a limited increase in grip strength. On the other hand, endurance (swimming time) was significantly enhanced in the BE-1X and BE-2X groups. These results are similar to those obtained after treatment with supplements rich in BCAA, such as chicken essence and whey protein [5,15]. BCAAs are not only basic muscle-building components but also participate in increasing protein synthesis in animals and humans [27,28]. Kato et al. reported that chronic BCAA supplementation led to increased performance in rats subjected to a swimming test [27]. On the basis of these findings, we suggest that BE supplementation can reduce swimming-induced muscle fatigue.

We previously found that supplementation with chicken essence after exercise could reduce plasma lactate and ammonia levels [15]. The concentrations of lactate, the product of glycolysis, is one of the important indicators of exercise fatigue. During strenuous exercise, muscles produce a considerable amount of lactate through anaerobic glycolysis [29]. The accumulation of lactate leads to pH reduction in muscle tissues, induces fatigue, and hampers exercise performance. As a result, rapid removal of lactate has been found to ameliorate fatigue [29]. In this study, BE-1X supplementation significantly decreased plasma lactate level, indicating that BE possibly inhibited the accumulation of this byproduct.

GLU is stored as glycogen, mainly in the liver and muscles. Glycogen metabolism is the most direct source of energy for muscles, and the volume of stored glycogen is a determining factor of fatigue [30]. During physical exercise, energy is derived from the breakdown of glycogen. As such, restoration of muscle glycogen is beneficial for prolonged exercise [31,32]. Indeed, decreased muscle glycogen level is a sensitive indicator of muscle fatigue. The BE-1X and BE-2X groups had dose-dependent increases in glycogen content in muscles, but not in the liver. This may directly increase exercise performance and reduce physical fatigue. Kato et al. reported that leucine-enriched essential amino acids modulate the recovery of glycogen content in the muscles after damage-inducing exercise [27]. In addition, leucine supplementation improved muscle performance and reduced depletion of muscle glycogen stores as compared with the mixture of BCAAs [28]. On the basis of these findings, we suggest that the reduction in muscle fatigue by BE supplementation might be related to leucine content, but further studies are needed to confirm this hypothesis.

Kim et al. found that supplementation with probiotics could increase intestinal short-chain fatty acid (SCFA) content [33]. SCFAs might affect the host energy balance and enhance athletic performance [34]. However, no significant differences in the concentrations of acetic acid, propionic acid, and butyric acid were observed among the study groups. These results may indicate that the ability of SCFAs to enhance exercise performance was not related to protein-rich supplements such as BE.

In summary, BE supplementation could significantly reduce physiological fatigue by decreasing serum lactate, maintaining GLU levels and preserving muscle glycogen content. These findings suggest that BE supplementation has a potential use for enhancing endurance or promoting physiological adaptation after intensive exercise training. Evidence obtained from clinical and biochemical evaluations also provides information regarding the safety of BE supplementation. Finally, we used GF mice to demonstrate that the anti-fatigue potential of BE supplementation could be related to nutritional composition but not the host gut microbiota.

## Figures and Tables

**Table 1:** Nutritional facts and total branched-chain amino acids from beef extract (BE).

Nutritional Facts	Content (/100 mL of BE)
Protein	7.7 g
Fat	0
Saturated fat	0
Trans fat	0
Carbohydrate	0
Sodium	0.094 g
Total calories (kcal)	30
Total BCAA (mg/100 g)	
Valine, leucine, and isoleucine	18.6

**Table 2:** General characteristics of the experimental groups.

Characteristics	Vehicle	BE-1X	BE-2X
Initial BW (g)	25.5 ± 1.3	25.8 ± 1.4	26.1 ± 1.4
Final BW (g)	25.6 ± 1.5	26.4 ± 1.3	26.2 ± 1.7
Skeletal muscle (g)	0.32 ± 0.03	0.33 ± 0.02	0.33 ± 0.03
Liver (g)	1.32 ± 0.10	1.32 ± 0.08	1.27 ± 0.09

The values are for *n* = 6–8 mice and presented as mean ± SD. Skeletal muscle mass is comprised of the gastrocnemius and soleus muscles in the posterior part of the lower legs.

**Table 3:** Biochemical analysis results of the BE supplementation groups at the end of the experiment.

Parameters	Vehicle	BE-1X	BE-2X
ALT (U/L)	48.2 ± 10.7	51.9 ± 9.9	55.2 ± 14.2
GLU (mg/dL)	233.0 ± 18.1	212.6 ± 19.8	213.5 ± 19.0
BUN (mg/dL)	24.4 ± 1.1	23.3 ± 1.0	23.9 ± 2.7
T-CHO (mg/dL)	143.8 ± 7.9	137.7 ± 7.3	156.7 ± 12.1
TG (mg/dL)	241.8 ± 67.1	195.7 ± 42.0	216.5 ± 25.3

The values are for *n* = 6–8 mice per group and presented mean ± SD. ALT, alanine aminotransferase; GLU, glucose; BUN, blood urea nitrogen; T-CHO, total cholesterol; TG, triacylglycerol.

**Table 4:** General characteristics and biochemical analysis results of the experimental groups.

Characteristics	GF-veh	GF-Alb	GF-BE
Initial BW (g)	26.8 ± 1.0	26.3 ± 1.8	27.8 ± 1.7
Final BW (g)	27.8 ± 0.7	28.4 ± 1.7	27.9 ± 0.9
Skeletal muscle (g)	0.30 ± 0.02	0.30 ± 0.02	0.29 ± 0.01
ALT (U/L)	46.2 ± 9.7	49.3 ± 13.5	55.0 ± 0.9
GLU (mg/dL)	221.0 ± 11.8	201.4 ± 28.1	211.9 ± 36.7
BUN (mg/dL)	23.9 ± 1.1	21.3 ± 3.0	22.9 ± 2.0
T-CHO (mg/dL)	193.8 ± 16.7	195.3 ± 22.1	195.9 ± 8.9
TG (mg/dL)	121.0 ± 25.1	99.2 ± 17.0	108.1 ± 13.7

Data are the mean ± SD for *n* = 5 mice. The skeletal muscle mass is comprised of the gastrocnemius and soleus muscles in the back part of the lower legs. ALT, alanine aminotransferase; BUN, blood urea nitrogen; T-CHO, total cholesterol; TG: triacylglycerol.

## References

[1] Yoshihara H., Wakamatsu J.-I., Kawabata F., Mori S., Haruno A., Hayashi T., Sekiguchi T., Mizunoya W., Tatsumi R., Ito T. (2006). Beef extract supplementation increases leg muscle mass and modifies skeletal muscle fiber types in rats. J. Nutr. Sci. Vitaminol..

[2] Hussain A., Olausson H., Nilsson S., Nookaew I., Khoomrung S., Andersson L., Koskela A., Tuukkanen J., Ohlsson C., Holmäng A. (2013). Maternal beef and postweaning herring diets increase bone mineral density and strength in mouse offspring. Exp. Biol. Med. (Maywood).

[3] Naclerio F., Larumbe-Zabala E., Ashrafi N., Seijo M., Nielsen B., Allgrove J., Earnest C.P. (2017). Effects of protein-carbohydrate supplementation on immunity and resistance training outcomes: A double-blind, randomized, controlled clinical trial. Eur. J. Appl. Physiol..

[4] Wyness L. (2016). The role of red meat in the diet: Nutrition and health benefits. Proc. Nutr. Soc..

[5] Chen W.C., Huang W.-C., Chiu C.-C., Chang Y.-K., Huang C.-C. (2014). Whey protein improves exercise performance and biochemical profiles in trained mice. Med. Sci. Sports Exerc..

[6] Burd N.A., Yang Y., Moore D.R., Tang J.E., Tarnopolsky M.A., Phillips S.M. (2012). Greater stimulation of myofibrillar protein synthesis with ingestion of whey protein isolate v. micellar casein at rest and after resistance exercise in elderly men. Br. J. Nutr..

[7] Robinson M.J., Burd N.A., Breen L., Rerecich T., Yang Y., Hector A.J., Baker S.K., Phillips S.M. (2012). Dose-dependent responses of myofibrillar protein synthesis with beef ingestion are enhanced with resistance exercise in middle-aged men. Appl. Physiol. Nutr. Metab..

[8] Fielding R., Riede L., Lugo J.P., Bellamine A. (2018). l-Carnitine supplementation in recovery after exercise. Nutrients.

[9] Brass E., Scarrow A., Ruff L., Masterson K., Van Lunteren E. (1993). Carnitine delays rat skeletal muscle fatigue in vitro. J. Appl. Physiol..

[10] Zainuddin Z., Newton M., Sacco P., Nosaka K. (2005). Effects of massage on delayed-onset muscle soreness, swelling, and recovery of muscle function. J. Athl. Train..

[11] Appell H.-J., Soares J., Duarte J. (1992). Exercise, muscle damage and fatigue. Sports Med..

[12] Behm D.G., Baker K.M., Kelland R., Lomond J. (2001). The effect of muscle damage on strength and fatigue deficits. J. Strength Cond. Res..

[13] Wan J.-J., Qin Z., Wang P.-Y., Sun Y., Liu X. (2017). Muscle fatigue: General understanding and treatment. Exp. Mol. Med..

[14] Hsu Y.-J., Huang W.-C., Lin J.-S., Chen Y.-M., Ho S.-T., Huang C.-C., Tung Y.-T. (2018). Kefir supplementation modifies gut microbiota composition, reduces physical fatigue, and improves exercise performance in mice. Nutrients.

[15] Huang W.-C., Lin C.-I., Chiu C.-C., Lin Y.-T., Huang W.-K., Huang H.-Y., Huang C.-C. (2014). Chicken essence improves exercise performance and ameliorates physical fatigue. Nutrients.

[16] Kim H.J., Lee Y.H., Kim C.K. (2007). Biomarkers of muscle and cartilage damage and inflammation during a 200 km run. Eur. J. Appl. Physiol..

[17] Waśkiewicz Z., Kłapcińska B., Sadowska-Krępa E., Czuba M., Kempa K., Kimsa E., Gerasimuk D. (2012). Acute metabolic responses to a 24-h ultra-marathon race in male amateur runners. Eur. J. Appl. Physiol..

[18] Lanier A.B. (2003). Use of nonsteroidal anti-inflammatory drugs following exercise-induced muscle injury. Sports Med..

[19] Ley R.E., Peterson D.A., Gordon J.I. (2006). Ecological and evolutionary forces shaping microbial diversity in the human intestine. Cell.

[20] Wilson B., Whelan K. (2017). Prebiotic inulin-type fructans and galacto-oligosaccharides: Definition, specificity, function, and application in gastrointestinal disorders. J. Gastroenterol. Hepatol..

[21] Zhang H., Li Y., Cui C., Sun T., Han J., Zhang D., Lu C., Zhou J., Cheong L., Li Y. (2018). Modulation of gut microbiota by dietary supplementation with tuna oil and algae oil alleviates the effects of d-galactose-induced ageing. Appl. Microbiol. Biotechnol..

[22] Bifari F., Ruocco C., Decimo I., Fumagalli G., Valerio A., Nisoli E. (2017). Amino acid supplements and metabolic health: A potential interplay between intestinal microbiota and systems control. Genes Nutr..

[23] Hooper L.V., Midtvedt T., Gordon J.I. (2002). How host-microbial interactions shape the nutrient environment of the mammalian intestine. Annu. Rev. Nutr..

[24] Hsu Y.J., Chiu C.C., Li Y.P., Huang W.C., Te Huang Y., Huang C.C., Chuang H.L. (2015). Effect of intestinal microbiota on exercise performance in mice. J. Strength Cond. Res..

[25] Saint-Maurice P.F., Laurson K., Welk G.J., Eisenmann J., Gracia-Marco L., Artero E.G., Ortega F., Ruiz J.R., Moreno L.A., Vicente-Rodriguez G. (2018). Grip strength cutpoints for youth based on a clinically relevant bone health outcome. Arch. Osteoporosis..

[26] Al-Sharif F.A.-G., Al-Jiffri O.H., El-Kader S.M.A., Ashmawy E.M. (2014). Impact of mild versus moderate intensity aerobic walking exercise training on markers of bone metabolism and hand grip strength in moderate hemophilic A patients. Afr. Health Sci..

[27] Kato H., Miura K., Suzuki K., Bannai M. (2017). Leucine-enriched essential amino acids augment muscle glycogen content in rats seven days after eccentric contraction. Nutrients.

[28] Campos-Ferraz P.L., Bozza T., Nicastro H., Lancha Jr A.H. (2013). Distinct effects of leucine or a mixture of the branched-chain amino acids (leucine, isoleucine, and valine) supplementation on resistance to fatigue, and muscle and liver-glycogen degradation, in trained rats. Nutrition.

[29] Cairns S.P. (2006). Lactic acid and exercise performance. Sports Med..

[30] Manabe Y., Miyatake S., Takagi M., Nakamura M., Okeda A., Nakano T., Hirshman M.F., Goodyear L.J., Fujii N.L. (2012). Characterization of an acute muscle contraction model using cultured C_2_C_12_ myotubes. PLoS ONE.

[31] Ivy J.L. (2004). Regulation of muscle glycogen repletion, muscle protein synthesis and repair following exercise. J. Sports Sci. Med..

[32] Hausswirth C., Le Meur Y. (2011). Physiological and nutritional aspects of post-exercise recovery. Sports Med..

[33] Kim H., Rutten N., Besseling-van der Vaart I., Niers L., Choi Y., Rijkers G., van Hemert S. (2015). Probiotic supplementation influences faecal short chain fatty acids in infants at high risk for eczema. Benef. Microbes.

[34] Chen Y.-M., Wei L., Chiu Y.-S., Hsu Y.-J., Tsai T.-Y., Wang M.-F., Huang C.-C. (2016). Lactobacillus plantarum TWK10 supplementation improves exercise performance and increases muscle mass in mice. Nutrients.

